# Carbohydrate vitrification in aerosolized saliva is associated with the humidity-dependent infectious potential of airborne coronavirus

**DOI:** 10.1093/pnasnexus/pgac301

**Published:** 2022-12-24

**Authors:** Marina Nieto-Caballero, Ryan D Davis, Eddie Fuques, Odessa M Gomez, Erik Huynh, Alina Handorean, Shuichi Ushijima, Margaret Tolbert, Mark Hernandez

**Affiliations:** Environmental Engineering Program, University of Colorado, Boulder, CO 80309, USA; Department of Chemistry, Trinity University, San Antonio, TX 78212, USA; Materials Reliability Department, Sandia National Laboratories, Albuquerque, NM 82123, USA; Department of Microbiology, Oregon State University, Corvallis, OR 97331, USA; Environmental Engineering Program, University of Colorado, Boulder, CO 80309, USA; Department of Chemistry, Trinity University, San Antonio, TX 78212, USA; Departments of Engineering Design and Society and Civil and Environmental Engineering, Colorado School of Mines, Golden, CO 80401, USA; Cooperative Institute for Research in Environmental Sciences and Department of Chemistry, University of Colorado, Boulder, CO 80309, USA; Cooperative Institute for Research in Environmental Sciences and Department of Chemistry, University of Colorado, Boulder, CO 80309, USA; Environmental Engineering Program, University of Colorado, Boulder, CO 80309, USA

**Keywords:** airborne, coronavirus, bioaerosol, efflorescence, persistence

## Abstract

An accepted murine analogue for the environmental behavior of human SARS coronaviruses was aerosolized in microdroplets of its culture media and saliva to observe the decay of its airborne infectious potential under relative humidity (RH) conditions relevant to conditioned indoor air. Contained in a dark, 10 m^3^ chamber maintained at 22°C, murine hepatitis virus (MHV) was entrained in artificial saliva particles that were aerosolized in size distributions that mimic SARS-CoV-2 virus expelled from infected humans’ respiration. As judged by quantitative PCR, more than 95% of the airborne MHV aerosolized was recovered from microdroplets with mean aerodynamic diameters between 0.56 and 5.6 μm. As judged by its half-life, calculated from the median tissue culture infectious dose (TCID_50_), saliva was protective of airborne murine coronavirus through a RH range recommended for conditioned indoor air (60% < RH < 40%; average half-life = 60 minutes). However, its average half-life doubled to 120 minutes when RH was maintained at 25%. Saliva microaerosol was dominated by carbohydrates, which presented hallmarks of vitrification without efflorescence at low RH. These results suggest that dehydrating carbohydrates can affect the infectious potential coronaviruses exhibit while airborne, significantly extending their persistence under the drier humidity conditions encountered indoors.

Significance Statement The infectious potential of airborne coronavirus depends on environmental factors, including relative humidity (RH) and the chemistry of its aerosol entrainment. Modern ventilation systems are primarily operated in RH ranges optimized for human comfort, which may significantly affect airborne virus persistence. Saliva can have a significant protective effect on airborne coronaviruses suspended in indoor air, which is amplified at lower RH levels. Carbohydrate was isolated as a protective agent of airborne coronavirus as it vitrified in salival microaerosols at low RH. Maintaining conditioned indoor air in a RH range between 60% and 40% is recommended, although many indoor atmospheres fall below this level during occupation cycles. Results suggest that maintaining increased RH indoors may affect the persistence of airborne coronavirus.

## Introduction

In the last two decades, novel *β-coronaviruses*—SARS-CoV-1 (2003), MERS-CoV (2012), and SARS-CoV-2 (2019)—have emerged into important zoonotic pathogens. Human transmission is suggested to take place through the following mechanisms: direct contact, indirect contact through fomites, via droplets, and through aerosols (1). The exact balance of infectious outcomes from these respective exposure routes remains unknown, although a dominance of aerosol-mediated exposure in indoor environments has been suggested (2, 3). While SARS-CoV-2-containing aerosols have been recently characterized in bench-scale laboratory chambers (4, 5) and cultured from air collected in health care settings (6, 7), the persistence of airborne *β-coronaviruses* remains poorly understood with respect to the chemical matrix in which it is co-aerosolized and aged in conditioned indoor air.

For successful airborne viral transmission to occur indoors, these virions must retain their infectious potential on timescales exceeding building air change rates (i.e., hours). In this context, the literature contains a modest body of classic literature that is often conflicting with respect to relative humidity (RH) effects as do some emerging reports on this topic (8–12). Regardless, there remains a paucity of mechanistic information describing how the infectious potential of airborne coronaviruses and their surrogates is influenced by environmental conditions, indoors and outdoors.

Certainly, some environmental factors influence the infective potential retained by airborne viruses in the built environment (4, 10, 13–17), most notably including temperature, RH, and light flux—acknowledging these variables are ostensibly controlled within relatively narrow ranges indoors. Some immediate chemical properties of the co-aerosol with which viruses are often introduced indoors (i.e., human respiratory and oral cavity fluids) have been implicated in protecting the viruses’ infectious potential (18–21). Among others, these properties include ionic strength, biopolymer associations (i.e., protein and carbohydrates), viscosity, and opacity, each of which can rapidly change as airborne microdroplets migrate through indoor spaces. Some synergy of these factors has been proposed in classic reviews (12), while others more recently demonstrated a relative increase in the infectious potential of zoonotic coronaviruses (or their surrogates), at RH extremes on surfaces (22). This synergy has also been leveraged to produce human measles vaccines by rapidly drying microdroplets containing high carbohydrate concentrations with live attenuated *morbillivirus*, which retains infectious potential for many months at low RH and room temperature (20°C) (23, 24). Many airborne coronavirus infectivity studies have been performed in laboratory settings, using common cell propagation media as a co-aerosolization solution (25). While these studies are an important step for understanding the persistence patterns of airborne coronaviruses, they may misestimate the infectivity potential decay of those virus-containing aerosols expelled by humans which are entrained with salivary or other respiratory fluids. Such fluids contain a milieu of biopolymers, which are hypothesized to be protective of enveloped viruses while airborne. These studies have suggested the importance of using artificial saliva as aerosolization media in viral infectivity studies, as it protects enveloped viruses and mimics a real setting (26). Laboratory studies using (artificial) respiratory solutions for infectivity assessment of airborne viruses commonly use small rotating cylindrical “drums” to minimize deposition losses. Many studies have used dry gelatin filters for rapid airborne virus sampling; however, dry filter collections may affect the infectious potential of enveloped virions due to the collection stresses associated with impaction, aging, and subsequent recovery (27, 28).

Many *β-coronaviruses* have remarkably similar physiology, including their glycoprotein receptors (S), membrane proteins (M), and envelope proteins (E), all of which are encoded by a 30 kilobase single-strand of positive-sensed RNA (+ssRNA). Within this subfamily, murine hepatitis virus (MHV) has gained acceptance as a model for the environmental persistence of related coronaviruses (29–31), as well as earned a distinction as a translational surrogate that can indicate the disinfection response of other human viruses (32).

This study observed time-resolved persistence patterns of airborne MHV, demonstrating a protective effect that artificial saliva confers to airborne coronavirus infectivity, at RHlevels germane to conditioned indoor air. This notably includes low humidity conditions like those sustained indoors in many temperate winter climates and arid regions (RH < 30%) (33). Airborne virus was collected by condensation capture to minimize collection stresses (34) during time-series experiments where MVH was allowed to age, suspended in air, for up to 2 hours. By periodically collecting airborne coronavirus with condensation capture, its persistence patterns could be observed at full scale in an environmentally controlled chamber (c.a. 10 m^3^) (35–37) through a broad range of RH conditions, with minimal sampling artifacts. The particle size distribution of the artificial saliva in which MHV was aerosolized, was controlled to correspond to those ranges expelled by hosts with respiratory tracts infected by influenza and rhinovirus (27). Optical patterns exhibited by microaerosolized saliva containing MHV were juxtaposed to RH-dependent viscosity behavior exhibited by mock respiratory solutions (38).

## Methods

### MHV propagation

MHV, strain A59 (MHV-A59, VR-764, ATCC, Manassas, VA, USA), was propagated in ≥95% areal confluent delayed brain tumor (DBT) cells, sourced from the University of Michigan Environmental Engineering laboratories, in T75 flasks in Dulbecco′s Modified—Eagle′s Medium (DMEM, 30–2002, ATCC, Manassas, VA, USA), including 2% fetal bovine serum (FBS, F2442, Sigma–Aldrich, St. Louis, MO, USA) with 1% antibiotic/antimycotic (A5955, Sigma–Aldrich, St. Louis, MO, USA) at 37°C and 5% CO_2_ for 48 hours, as previously described by Leibowitz and coworkers (39).

### Chamber experiments with MHV in propagation media

An environmental chamber with temperature and RH control, well-mixed, particle-free (below two particles/L), and with a volume of c.a. 10 m^3^ was used in this study. Each chamber experiment was performed in triplicate on different days, separated by at least a week. On any experiment day, T75 flasks containing 48 hours postinfection MHV were centrifuged at 3,000 x *g* for 5 minutes to remove any cell debris, and 8 mL of the supernatant were nebulized in the chamber with a 6-jet collison nebulizer (CH Technologies, Westwood, NJ, USA) at 20 psig for 8 minutes and 15 seconds with HEPA-filtered, compressed breathing air (AI B300, Airgas, Radnor, PA, USA). Virus-containing aerosol was subsequently collected for 5 minutes at a flow rate of 8 L/min at times 0, 10, 20, 40, 60, 90, and 120 minutes post nebulization with a BioSpot-VIVAS condensation growth tube sampler (Aerosol Devices, Fort Collins, CO, USA) collecting directly into 2 mL DMEM with 2% FBS including 1% antibiotic/antimycotic. The temperature array of the BioSpot-VIVAS condensation growth tube was set to 5, 45, 10, 25, and 12°C for the conditioner, initiator, moderator, nozzle, and sample temperatures, respectively.

### Chamber experiments with MHV in artificial saliva

For experiments aerosolizing MHV entrained in artificial saliva, sterile, research-grade medical saliva (1700–0305, Pickering Laboratories, Mountain View, CA, USA) was used according to the following protocol. The supernatant from three MHV-infected T75 flasks was added to two 30 kDa cutoff Amicon Ultra 15 mL Centrifugal Filters (UFC9030, Sigma–Aldrich, St. Louis, MO, USA) and was centrifuged for 40 minutes at 4,000 x *g*. The retentates were resuspended in a total volume of 10 mL of artificial saliva, 8 mL of which were nebulized in the environmentally controlled chamber, previously described, with a 6-jet collison nebulizer under otherwise identical conditions to propagation media (DMEM with 2% FBS).

### Infectivity assay

The Reed–Muench method (41) was used to determine viral infectivity in terms of median tissue culture infectious dose (TCID_50_) in 96-well plates with 100 μL of sample per well, as described by Leibowitz and coworkers (39), was modified to include 10% antibiotic/antimycotic, to avoid any bacterial or fungal contamination following full-scale aerosol collection. Infectivity assays for identification of cytopathic effect were performed in 96-well plates, which were assessed with a Celígo Imaging Cytometer (Nexcelom Bioscience, Lawrence, MA, USA) 48 hours postinfection.

### Airborne viral genome quantification

Volumes of 200 µL of samples collected with the BioSpot-VIVAS were immediately added to 200 µL DNA/RNA Shield (R1100, Zymo Research, Irvine, CA, USA) and stored overnight at −20°C. RNA was extracted with the Quick-RNA Viral Kit (R1035, Zymo Research, Irvine, CA, USA) according to the manufacturer's instructions and resuspended in 15 µL of ultrapure molecular biology grade water. The extracted RNA was immediately retrotranscribed with the RevertAid RT Reverse Transcription Kit (K1691, Thermo Fisher Scientific, Waltham, MA, USA), following the manufacturer's protocol, using 10 µL of the extracted RNA and 2 µL of random hexamer primers provided by the kit.

Complementary DNA (cDNA), obtained from retrotranscription, was subsequently amplified, and quantified by quantitative polymerase chain reaction (qPCR) in a QuantStudio 3 real-time PCR system (Applied Biosystems, Foster City, CA, USA) with a TaqMan probe. The forward primer was previously described by Besselsen and coworkers (40), while the reverse primer and probe, listed in Table 1, were specifically designed for this study. An amplicon of 108 base pairs, belonging to the most highly conserved gene region in murine coronaviruses (42), which encodes for the membrane protein (M), was used.

**Table 1.** Primer and probe sequences for RT-qPCR of MHV-A59.

	Sequence (5’ → 3’)
**Forward primer** (40)	GGAACTTCTCGTTGGGCATTATACT
**Reverse primer**	ACCACAAGATTATCATTTTCACAACAT
**Probe**	56-FAM/TTCGGTTACACGAGCCGTAGCATG/3BHQ_1

qPCR assay was carried out with the following conditions: a final concentration of 1X iTaq Universal Probes Supermix (1725131, Bio-Rad, Hercules, CA, USA), 0.15 µM forward primer, 0.15 µM reverse primer, 0.1 µM probe, 3.5 mM MgCl_2_ (includes MgCl_2_ contained in the master mix) and 1 µL of cDNA template in a total volume of 20 µL. The reaction was performed in standard mode with an initial step to activate the polymerase at 95°C for 5 minutes, followed by a cycle that was repeated 40 times using a first stage of DNA denaturization at 95°C for 15 seconds and a second stage of annealing/extension and plate reading at 60°C for 1 minute. In order to determine the initial amount of template in each sample, a standard was developed circumscribing eleven, 10-fold serial dilutions containing between 10^10^ to 1 copies/μL of the synthetic amplicon (Ultramer DNA Oligonucleotides, Integrated DNA Technologies, Coralville, IA, USA).

#### Characterization of particle deposition

Throughout the course of these chamber experiments, a Wideband Integrated Bioaerosol Sensor (InstaScope, Boulder, CO, USA) was used to monitor aerosol concentrations in the chamber with optical diamters between 0.5 to 10 μm, in real time. The associated particle deposition was calculated from the changes in airborne particle inventories for every bioaerosol chamber challenge in this study. The net deposition is reported as an averaged particle number decay rate for each RH and co-aerosol condition; reported as a mean (*k_Net Deposition_*), these rates are compiled along with their corresponding standard deviations in the Supplementary Material Table S1.

#### Isolating airborne viral decay

The airborne virus decay reported in this study quantitatively accounted for particle deposition (> 0.5 μm) in the chamber. This effectively isolated the declines of airborne viral RNA and culturing potential from any surface losses, including particle deposition on the walls and floor. The deposition rate (*k_Net Deposition_*) for each environmental condition, acquired from the InstaScope particle monitoring, was subtracted from the respective airborne decay of TCID_50_/m^3^ and RNA/m^3^ and is reported here (Table 2) as effective mean decay rates (*k_TCID50_* and *k_RNA_*, respectively).

**Table 2.** Effective decay rates (k, h^−1^) and SDs of airborne MHV infectivity (TCID_50_) and genetic material (RNA copies), and average effective half-life (h).

	k_TCID50_ (h^−1^)	k_RNA_ (h-1)	Average t_1/2 (Eff)_ (h)
DMEM 25%	0.677 ± 0.250	0.577 ± 0.183	1.024
Saliva 25%	0.346 ± 0.06	0.546 ± 0.035	2.003
DMEM 40%	0.845 ± 0.035	0.625 ± 0.069	0.820
Saliva 40%	0.788 ± 0.151	0.928 ± 0.386	0.880
DMEM 60%	1.538 ± 0.707	1.298 ± 0.624	0.451
Saliva 60%	0.632 ± 0.092	0.772 ± 0.060	1.097

These include results from environmental chamber experiments at three RH levels, and are corrected for net particle losses on chamber surfaces. Three chamber runs were performed on separate days for each condition.

### Half-life observations

Effective half-life (t_1/2_) values were calculated using TCID_50_/m^3^ results obtained from time-series collections in the environmental chamber which accounted for aerosol deposition as described above. First, decay rates were calculated for each combination of RH and co-aerosolization conditions. These decay rates, or *k_TCID50_* were obtained from classic, batch first-order reaction rates in completely mixed conditions (Equations 1 and 2), where *t* is time in hours, *C_i_* is concentration at the beginning of the experiment in units of TCID_50_/m^3^, *C* is concentration at any given time and *k* is the exponential decay rate in units of h^−1^. Analogous decay rates were estimated for the qPCR time-series results obtained (*k_RNA_*) by changing the concentration units of Equations 1 and 2 to RNA copies/m^3^.

(1) \begin{eqnarray*} \mathit{ C} = {\mathit{ C}_i}{e^{ - kt}}, \end{eqnarray*}

(2) \begin{eqnarray*} k = \left( {\frac{1}{\mathit{ t}}} \right)\,\ln \left( {\frac{{{\mathit{ C}_i}}}{\mathit{ C}}} \right). \end{eqnarray*}

### Protein concentration

Protein concentrations for the aerosolization solution in each individual chamber experiment (i.e., MHV in viral propagation media and MHV in artificial saliva) were measured in a Qubit 4 fluorometer with Qubit Protein Assay Kit (Invitrogen, Carlsbad, CA, USA).

#### Particle size distribution

A Micro-Orifice Uniform Deposition Impactor (MOUDI, TSI Incorporated, Shoreview, MN, USA) was used for size-fractionated particle characterization (43) in separate chamber runs where humidity was controlled at 25% and 60% RH.

##### Gelatin-DMEM MOUDI impaction plate preparation

Heavy-duty aluminum foil was punched into 47-mm-diameter circles. These were attached on respective MOUDI impaction plates, then sterilized with 70% ethyl alcohol and illuminated under a 30-watt UV lamp (c.a. 40 cm from the source) in a type A2 biosafety cabinet for 10 minutes (Purifier Logic +, Labconco, Kansas City, MO, USA).

Six gelatin filters with 47 mm diameter (12602—47—ALK, Sartorius, France) were placed with sterile tweezers in a 50 mL centrifuge conical tube containing 12 mL DMEM. The mixture was warmed and mixed at 37°C until completely dissolved; 5 mL of this gelatin-DMEM mixture was evenly distributed on sterile aluminum surface plates fitted for each of the MOUDI stages described above. These gel-coated inserts were then subsequently placed in sterile Petri dishes, allowed to solidify, then wrapped with sterile plastic covers to minimize drying; these were stored overnight at 4°C then used for size-segregating virus aerosol collection, the following day.

##### MOUDI sampling in chamber experiments

The MOUDI sampler was operated at a flow rate of 30 L/minute, as verified by an inline rotameter, and operated for five continuous minutes. The preprepared gelatin-DMEM plates were placed on the top 11 of the MOUDI, which corresponded to interval nominal size cuts, from top to bottom, of >18, 10 to 18, 5.6 to 10, 3.2 to 5.6, 1.8 to 3.2, 1.0 to 1.8, 0.56 to 1.0, 0.32 to 0.56, 0.18 to 0.32, 0.1 to 0.18, and 0.056 to 0.1 µm. On the lower stage, corresponding to any particle smaller than 0.056 μm, a sterile 37 mm diameter quartz membrane (Pallflex Tissuquartz, Pall Corporation, Port Washington, NY, USA) was used.

Volumes of 8 mL MHV in artificial saliva was nebulized for 30 seconds at 14 kPA as previously described. In separate trials, the aerosolized virus was allowed to age for 20 minutes at the RH extremes circumscribing these experiments (25% and 60% RH).

##### Recovering MHV RNA from MOUDI stages

Eleven 50 mL sterile centrifuge tubes with 10 mL DMEM were placed in a water bath at 37°C to warm the solution. In their entirety, the gelatin-DMEM films and their aluminum substrates were removed from each of the MOUDI stages and immediately placed in a biosafety cabinet. Impaction plates were disassembled, and gelatin surfaces with accompanying aluminum foil were carefully placed with sterile tweezers in sterile 50 mL centrifuge tubes containing 10 mL of warm DMEM. These were placed on an orbital shaker at 37°C and 170 rpm until the gelatin was completely dissolved (c.a. 10 minutes).

The solubilized gelatin was subsequently vortexed for 10 seconds, and 100 µL were transferred to 1.5 mL microcentrifuge tubes containing 100µL genomic preservative (DNA/RNA Shield, Zymo Research, Irvine, CA, USA). RNA was extracted following the Quick-RNA Viral Kit (R1035, Zymo Research, Irvine, CA, USA) protocol, and was eluted into 15 μL molecular biology grade water and processed for RT-qPCR, as previously described. The RT-qPCR recovery from each MOUDI stage was compared to its counterparts at the RH extremes circumscribing this study (25% < RH < 60%).

#### Characterization of aerosol phase states

The phase behavior of artificial saliva aerosols entraining MHV was characterized using the dual-balance quadrupole electrodynamic balance (DBQ-EDB) technique (44, 45). Hygroscopic growth was studied using a single-particle approach, whereas the viscous properties of artificial saliva were elucidated with a dual-particle approach. Regardless, levitated aerosol particles were generated from stock propagation media that was diluted 3-fold in filtered Millipore water using an on-demand piezoelectric droplet dispenser (MJ-ABP-060, Microfab, Plano, TX, USA). Levitated particles were injected into the DBQ-EDB through an induction electrode (≤ ±250 V_dc_, typical) that was axially confined in a quadrupole cavity (±1000 V_ac_, 150 to 1300 Hz). Counterbalance electrodes countered the force of gravity and the drag force from a humidified nitrogen gas flow (400 sccm, typical).

##### Single-particle assessment

Hygroscopic particle behavior was characterized by levitating single particles and tracking the relative mass as the RH was varied. The mass of the levitated particle is directly proportional to the counterbalance voltage, *V*_CB_, under zero flow. For dehumidification, the initial RH was approximately 85% and was subsequently lowered to 0% in 5% increments. The same particle was then studied upon humidification by raising the RH. The mass growth factors (MGF) were calculated relative to its mass at 0% RH according to the following relationship

(3) \begin{eqnarray*} MGF = \frac{{{V_{CB}}\left( {RH} \right)}}{{{V_{CB}}\left( {0\% \,RH} \right)}}. \end{eqnarray*}

If present, efflorescence is observable as an abrupt mass drop due to particle-phase water loss, concomitant with distinct changes in the far-field laser scatter patterns (45).

##### Dual-particle studies

The viscous properties of artificial saliva containing coronavirus as aerosolized here were studied in the DBQ-EDB, using methods described extensively elsewhere (44, 45). Two oppositely charged saliva particles were simultaneously levitated at a fixed RH and then subsequently merged after conditioning at constant RH for 10 minutes. Using brightfield imaging, the characteristic lengths of merging dimers were monitored and measured as a function of time, where the aspect ratio is defined by the long axis relative to the short axis of the merged composite. For viscous Newtonian fluids, exponential decreases in aspect ratio juxtaposed to a time constant is directly proportional to viscosity. While non-Newtonian fluids exhibit more complex coalescence and may not completely merge; regardless, both cases were considered here.

## Results

### Half-life of airborne MHV

**Fig. 1.**
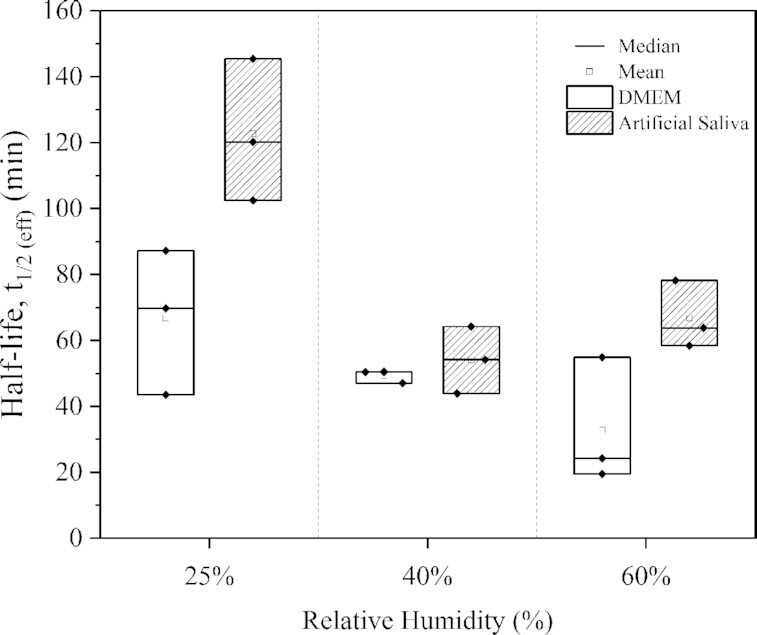
Effective half-life of airborne mouse hepatitis virus (MHV) based on TCID_50_ recovery from the air contained in a full-scale environmentally controlled chamber (c.a. 10 m^3^) at different RH levels; when aerosolized in culture media (DMEM with 2% FBS) (□); and, under otherwise identical conditions in artificial saliva (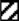).

As judged by patterns of TCID_50_ decay, the airborne half-life of MHV infectious potential was determined from six independent aerosolization trials. These experiments were performed in triplicate for each independent condition, where three different (constant) RH levels relevant to conditioned air for indoor environments (i.e., 25%, 40%, and 60% RH), were tested on separate days at constant temperature (22°C). Experimental scenarios were defined by the following co-aerosol that contained the same amounts of infection-competent MHV: (i) viral propagation media (DMEM with 2% FBS); or (ii) artificial saliva, that once aerosolized were contained at a constant RH level for 2 hours. The experimental isolation of these co-aerosols revealed a marked protective effect of saliva entrainment, regardless of RH (Fig. 1).

The media used to propagate MHV (DMEM with 2% FBS) contained an average of 8.2 mg/L of protein when used to aerosolize the viral particles, while the artificial saliva aerosolizing MHV contained 31.5 mg/L protein in addition to 25 g/L of methylcellulosic carbohydrates. As judged by one-way analysis of variance (ANOVA), saliva entrainment had a significant effect on the airborne survival of MHV (*P*value = 0.031, Fig. 1), regardless of RH level. At low RH, airborne MHV retained its infective potential significantly longer (*P* value = 0.011) than that at higher RH levels (i.e., 40% and 60% RH). Based on Tukey's test (95% CI), the mean MHV half-life at 25% RH significantly differed from either of the higher RH levels tested (40% and 60%), yet no significant difference was observed between 40% and 60% RH levels (*P* value = 0.854).

### Airborne virus inactivation, particle size distribution, and deposition potential

Metrics describing the airborne behavior of MHV in this full-scale chamber are summarized in Table 2. A log transformation was performed on the time series data recovered, and regressions elucidated significant differences in the decay patterns of MHV infectious potential (TCID_50_) as well as with its airborne RNA (RT-qPCR). All biological decay rates reported account for particle deposition under the environmental condition operated. As judged by RNA gene copy numbers and TCID_50_, degradation rates of MHV's genetic material were statistically indistinguishable from the rate that airborne MHV lost its infectious potential (*P* value = 0.956), yet gene copy numbers were consistently three orders of magnitude higher than the TCID_50_ at each corresponding time point measured (Table 3).

**Table 3.** Average ratio of airborne infective virions to total RNA gene copies following 8.25 minutes of MHV nebulization.

RH (%)	DMEM (TCID_50_/RNA)_t = 0_	Artificial saliva (TCID_50_/RNA)_t = 0_
**25**	4.66E + 03	4.99E + 03
**40**	4.68E + 03	8.53E + 03
**60**	3.79E + 03	8.59E + 03

Three orders of magnitude difference were consistently found for all the tested environmental conditions.

**Fig. 2.**
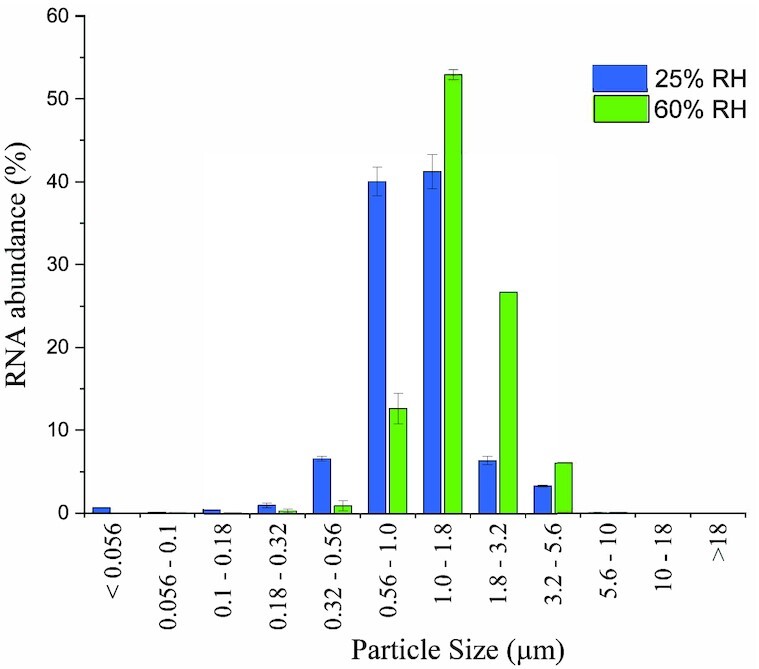
Particle size distribution of MHV in saliva when aerosolized at 25% RH (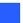) and 60% RH (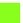) as judged by RNA relative abundance in 11 MOUDI stages. Airborne MHV was aged for 20 minutes at constant RH in the chamber, and RNA was assessed with RT-qPCR.

As judged by segregation of RNA across the stages of the MOUDI impactor, the airborne MHV virus was largely constrained in a particle size distribution between 0.5 and 3.5 μm (D_50_) in artificial saliva, regardless of the boundaries of RH observed here (Fig. 2). At low RH (25%), approximately 85% of the particles had a mean aerodynamic diameter in the range between 0.56 and 1.8 µm, while at the highest RH tested (60%), approximately 80% of the particles were found in the range between 1.0 and 3.2 µm.

The relatively small shift in particle size distribution observed is likely due to differences in evaporative losses at the respective humidity levels, acknowledging these airborne particles were near an equilibrium condition after aging 20 minutes in the humidity-controlled air, prior to their collection. Despite this shift in particle size distribution, nearly all (c.a. > 97%) MHV RNA-containing particles were found in a size range previously associated with human viral respiratory loads (0.5 to 5.0 μm) during the timeframe of these experiments (46, 47), regardless of the RH level.

Based on the particle size distribution aerosolized, the settling velocities corresponding to the aerosolized particles in these experiments were found to range between 0.017 and 1.15 m/h for the smallest and largest particles containing viral RNA, respectively. To maintain a well-mixed environment, three low-power fans (4 W) were placed at the bottom of the environmental chamber. The effective decay of airborne MHV infectivity observed, accounted for losses due to virus-containing particle deposition, which was effectively constant across all experiments regardless of RH, in the time frames observed (c.a. 2 hours).

**Fig. 3.**
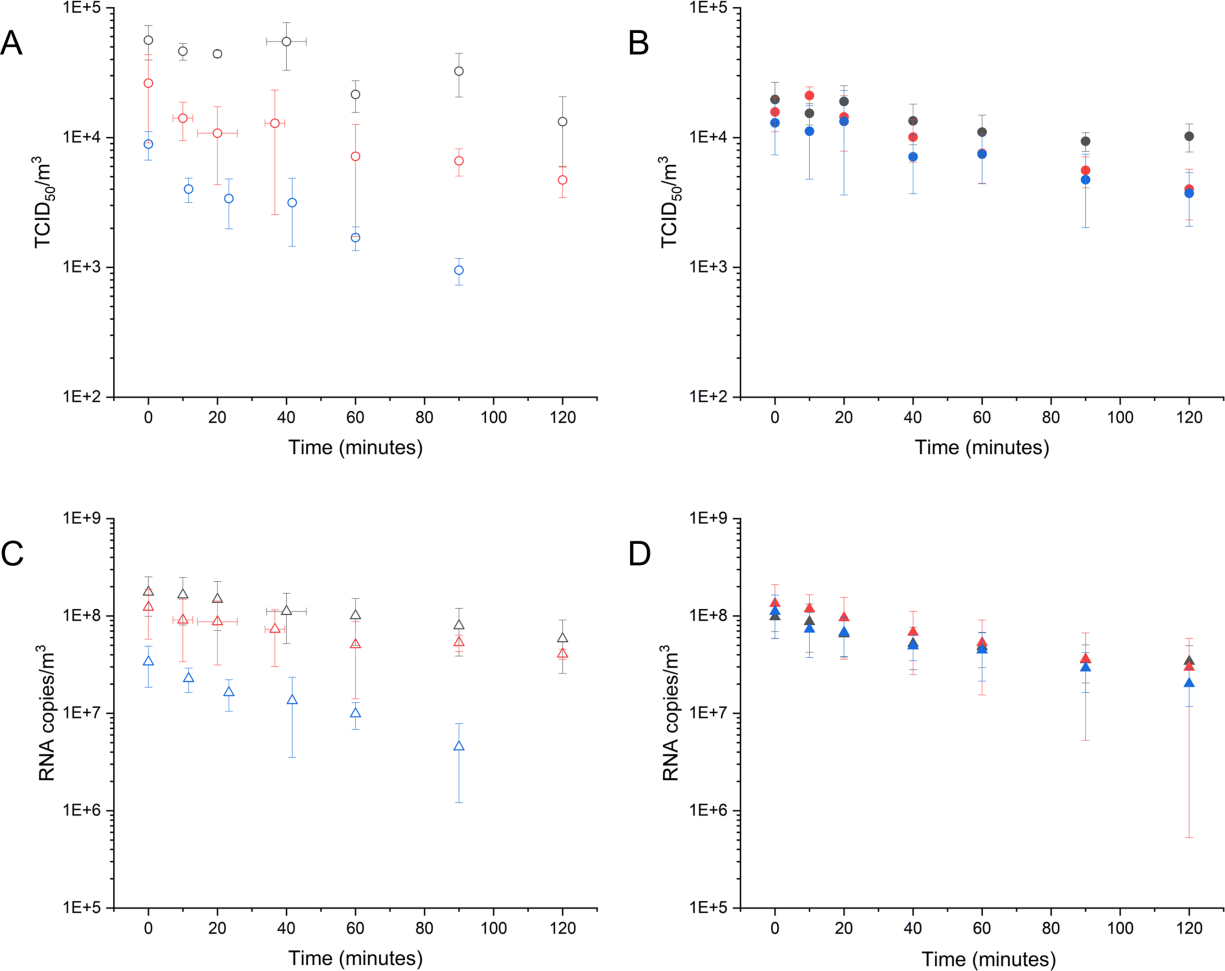
Time-series (minutes) showing averaged airborne MHV recovery as judged by TCID_50_/m^3^ [A and B panels (circles)] and RNA copies/m^3^ [C and D panels (triangles)] in the environmental chamber maintained at constant RH for up to 120 minutes. A and C panels (open icons) present airborne MHV recovery aerosolized in DMEM; B and D panels (filled icons) present MHV recovery aerosolized in artificial saliva. Gray icons represent 60% RH conditions; red icons represent 40% RH; and blue icons represent 25% RH conditions. Error bars represent pooled SDs from independent chamber experiments performed in triplicate.

As shown in Fig. 3, and summarized in Table 3, a significant difference between airborne TCID_50_/m^3^ levels and RNA copies/m^3^ was observed at all times of the experiments (*P* value < 10^−10^).

### Humidity-dependent phase behavior of aerosolized artificial saliva

In the complete absence of virus, airborne DMEM droplets have been reported to exhibit humidity-dependent phases, where a gelatinous transition occurs at or near 55% RH and some evidence of efflorescence presenting near 35%. Here we report a parallel characterization of DMEM with 2% FBS and artificial saliva microdroplets containing significant amounts of murine coronavirus, through a broad humidity range. Under these conditions, saliva presented viscosity changes that were significantly greater than water or DMEM with 2% FBS.

**Fig. 4.**
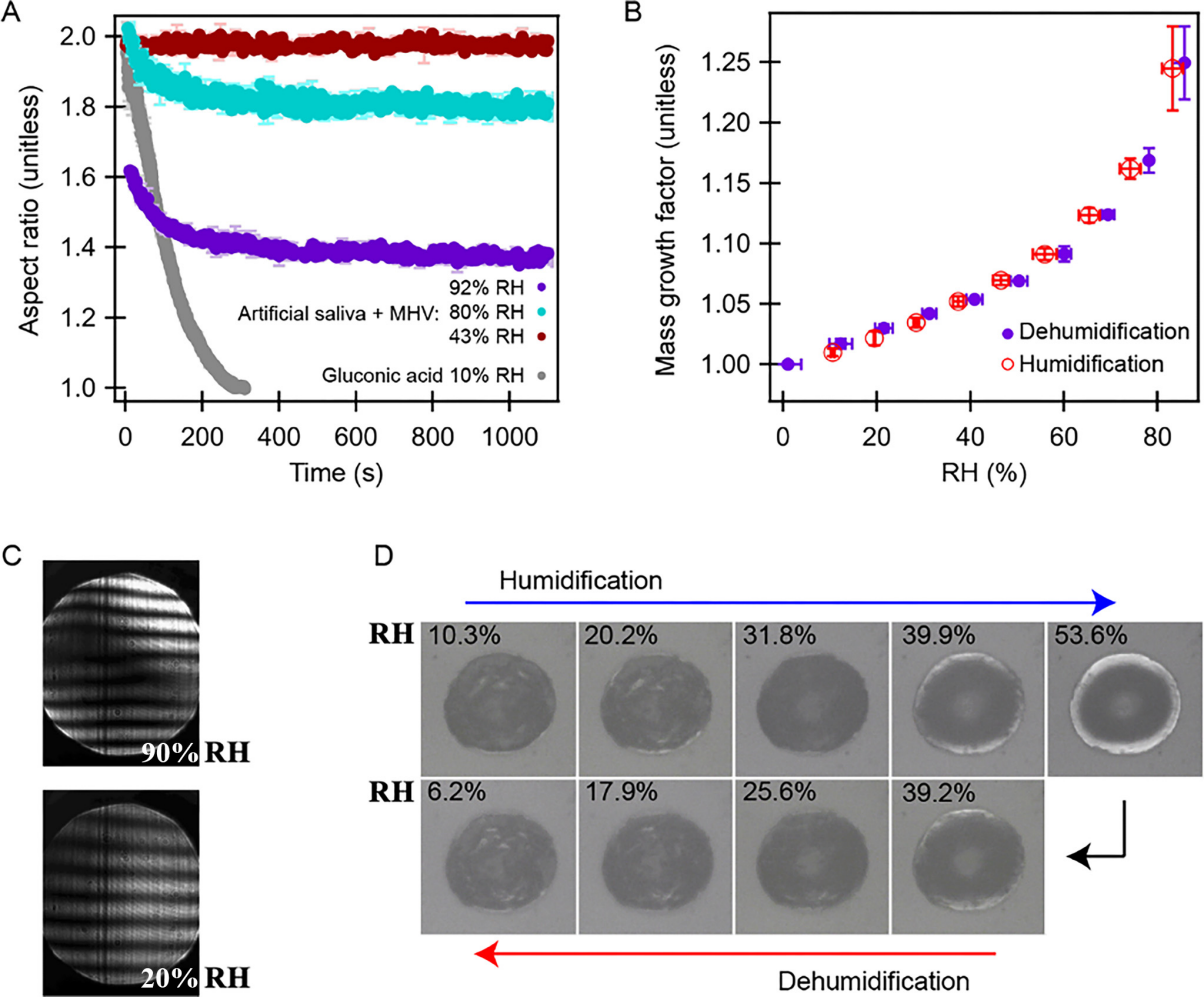
Microscopic observations of the humidity-dependent phase change behavior of levitated (A) to (C) and immobilized (D) saliva microdroplets containing murine coronavirus: (A) Coalescence kinetics of levitated, airborne saliva microdroplets juxtaposed to gluconic acid; (B) mass growth of levitated, airborne saliva microdroplets (c.a. 10 μm); (C) far-field laser scatter images of single levitated, airborne saliva microdroplets showing Mie scattering representative of spherical liquid or vitrified droplets; and (D) brightfield phase contrast microscope photographs typical of immobilized saliva microdroplets matriculating through humidification and dehumidification cycles (1000x, characteristic length, c.a. 40 μm) showing no phase transitions.

As shown in Fig. 4A, levitated saliva aerosol particles containing coronavirus present a variable, incomplete coalescence behavior at relatively high humidity levels (RH ≥ 80%), when juxtaposed to otherwise particles suspended in a lower humidity atmosphere (c.a. 40%) and gluconic acid at low humidity (RH = 10%). For comparison, the coalescence of binary gluconic acid (water and gluconic acid) at 10% RH demonstrates homogenously mixed phases associated with Newtonian fluid behavior, which serves as a control. The constant aspect ratio presented by saliva microdroplets containing virus at 43% RH is consistent with non-Newtonian fluid behavior, tending toward multiphase system be-havior.

As shown in Fig. 4B and C, artificial saliva droplets hosting coronavirus become obviously rigid in response to RH dropping below 40%; in this lower RH range, there is a substantial reduction in particle mass growth rate (d[MGH]/d[RH]) (Fig. 4B); and, no changes in far-field laser scattering patterns (Fig. 4C). As represented in Fig. 4D, immobilized saliva droplets containing coronavirus presented no optical evidence of hysteresis through multiple humidification and dehumidification cycles. The composition of the microdroplets observed, their non-Newtonian behavior (d[AR]/d[RH] and d[MGH]/d[RH]) and the lack of efflorescence suggest that the artificial saliva observed here, exists in a gelatinous state, which vitrifies in response to RH depression.

## Conclusions and discussion

In this study, the infectivity of airborne coronavirus was characterized and controlled in a respirable particle size range at different RH levels in a full-scale experimental setting. When isolated as a process variable, RH appeared to play a significant role in the persistence of airborne coronavirus infectivity, as has been shown in parallel experimental studies on surfaces (13, 22) and other (cell growth) media-containing aerosols (14–16). This condition was amplified by entrainment in an artificial saliva within which the carbohydrates appeared to experience vitrification as humidity dropped to 25%.

A pattern defined by higher effective TCID_50_ half-life (t_1/2_) emerged at or below 25% RH from experiments performed with artificial saliva, which has been reported in a laboratory study of other enveloped bacteriophages (Phi6) immobilized on surfaces (18). In this study, the effective decay of airborne MHV infectious potential, significantly decreased at low RH levels (25% RH) when compared to ranges recommended for conditioning indoor air (40% ≤ RH ≤ 60%). This pattern was recently reported for other *β-coronaviruses* on surfaces, as well as other enveloped viruses suspended in aerosols (10, 22, 48). This observation has implications for areas with low RH climate patterns, which in this context may benefit from controlling RH levels in the range above 40% in order to lower the infectious potential of airborne coronaviruses or other enveloped airborne viral pathogens entrained in saliva microaerosols indoors.

As judged by TCID_50_, saliva was found to be an important factor associated with the persistence of airborne MHV infectious potential. Aerosol microdroplets associated with infectious aerosol transmission have been reported to contain either or both saliva and respiratory fluid, with concentrations of protein ranging between 0.03 and 85 g/L (49–51). In previous studies, the protein content (18, 20) of saliva has been associated with efflorescence and/or a protective effect for airborne enveloped viruses (19). Further, purposeful aerosol drying with selected carbohydrates has also been shown to stabilize pathogenic human viruses while airborne (i.e., measles) (23, 24). In the complete absence of efflorescence, carbohydrate (methylcellulose) vitrification in artificial saliva was implicated here by clear, independent lines of physical and optical evidence. The juxtaposition of the aerosolization media used in these experiments (DMEM with 2% FBS vs. artificial saliva) isolated a protective effect with respect to conventional cell growth media, regardless of the RH condition tested here. This protective effect may be due to the increased viscosity of artificial saliva relative to its propagation media. These results suggest that use of artificial saliva, and/or other respiratory reagents that model the properties of actual mammalian body fluids can provide meaningful advancements toward understanding mechanisms that support the persistence of airborne virus infectivity in the built environment.

The protective effect of viscous phase states has been previously suggested (45). Here, we present experimental evidence isolating carbohydrate vitrification as an enveloped virus support mechanism by using artificial saliva, including model polysaccharides as the dominant biopolymer (methylcellulose). In this context, DMEM with 2% FBS, which is often used as a medium to propagate and aerosolize Corona- and other mammalian viruses, has been shown to remain as a relatively nonviscous fluid until undergoing a gelatinous transition near 55% RH (45). This gel transition could help explain the increase in MHV infectivity between 60% and 40% RH when DMEM with 2% FBS was the sole aerosolization media containing airborne coronavirus. The further infectivity increase observed as RH dropped from 40% to 25% RH is coincident with the previously observed efflorescence threshold at 35% where DMEM alone has been used (45). However, when artificial saliva was the aerosolization medium, the infectivity of MHV was elevated at all RH levels compared to DMEM with 2% FBS under otherwise identical conditions. These results suggest that the increasing viscosity of artificial saliva undergoing carbohydrate vitrification is a mechanism that can contribute to enhanced virus survival at lower humidity levels. This protective effect may be enabled by hindering diffusion and thus the decomposition of viral biopolymers (52). In response to carbohydrate vitrification, viscosity increases can also influence protein stability (53). Thus, protective effects realized from vitrification and/or efflorescence may be associated with stabilizing the virus’ surface proteins.

Significant differences between TCID_50_/m^3^ levels and RNA copies/m^3^ suggests that RT-qPCR overestimates infectious airborne viral exposure. This is likely attributed to the distribution of airborne RNA pools, including the following: RNA within virions of infective viruses, RNA in virions with no infective capabilities, and as a pool of RNA associated with other cellular detritus. As judged by RT-qPCR, this discrepancy was demonstrated to be approximately three orders of magnitude higher when compared to viral infective aerosol particles recovered from this full-scale chamber, accounting for nominal deposition. As RNases are present in microorganisms, human skin, and the environment (54–56); more genetic material degradation is potentially occurring outside of laboratory settings, which implies that degradation of viral RNA could potentially occur in indoor or outdoor environmental settings.

Moreover, a nebulizer generating virus-containing particles in a relatively narrow aerosol size distribution (0.5 < D_50_ < 5.0 μm) was used in an attempt to represent those particles expelled from human respiration, including common vocal activity. Future studies might benefit from a nebulization with a wider polydispersed particle size distribution, even though larger diameter particles would deposit more rapidly than those observed here.

This study limited its humidity range to that of conditioned indoor air commonly cycled through modern buildings in developed countries. Future persistence studies could be expanded to include environments with more extreme humidity conditions—including RH levels below 25% [e.g., (sub)arctic and desert regions], as well as higher RH levels relevant to more humid areas with different traditions of occupation patterns (e.g., ≥ 80% RH).

## Supplementary Material

pgac301_Supplemental_File

## Data Availability

Data generated by this study and used in this paper can be found in the repository on Zenodo: http://doi.org/10.5281/zenodo.7430441.
