# Supplementary material for: Carbohydrate vitrification in aerosolized saliva is associated with the humidity-dependent infectious potential of airborne coronavirus
Source: PNAS Nexus. 2022 Dec 24;2(2):pgac301. doi: 10.1093/pnasnexus/pgac301 (PMC9896139; doi:10.1093/pnasnexus/pgac301)
Supplement: pgac301_Supplemental_File [file pgac301_supplemental_file.docx]

**Supplementary Information:**

**Table S1.** Total net deposition rates from InstaScope data for each experimental condition tested in the study.

| **Experimental condition** | **k_Net Deposition_ (h^-1^)** |
| --- | --- |
| DMEM 25% | 0.003 ± 0.001 |
| Saliva 25% | 0.014 ± 0.003 |
| DMEM 40% | 0.015 ± 0.002 |
| Saliva 40% | 0.012 ± 0.008 |
| DMEM 60% | 0.022 ± 0.012 |
| Saliva 60% | 0.008 ± 0.003 |
